# Variations in DNA methylation and the role of regulatory factors in rice (*Oryza sativa*) response to lunar orbit stressors

**DOI:** 10.3389/fpls.2024.1427578

**Published:** 2024-11-14

**Authors:** Xiaohui Du, Yan Zhang, Meng Zhang, Yeqing Sun

**Affiliations:** Institute of Environmental Systems Biology, College of Environmental Science and Engineering, Dalian Maritime University, Dalian, China

**Keywords:** DNA methylation, methylation regulatory factors, miRNAs, lunar orbit stressors, spaceflight, single sample analysis, rice (*Oryza sativa*)

## Abstract

Deep space flight imposes higher levels of damage on biological organisms; however, its specific effects on rice remain unclear. To investigate the variations in DNA methylation under deep space flight conditions, this study examined rice seeds carried by Chang’e-5. After 23 days of lunar orbital flight, the samples were planted in an artificial climate chamber and subjected to transcriptome and DNA methylation sequencing during the tillering and heading stages. The methylation patterns in the rice genome exhibited variability in response to lunar orbital stressors. DNA methylation alters the expression and interaction patterns of functional genes, involving biological processes such as metabolism and defense. Furthermore, we employed single-sample analysis methods to assess the gene expression and interaction patterns of different rice individuals. The genes exhibiting changes at the transcriptional and methylation levels varied among the different plants; however, these genes regulate consistent biological functions, primarily emphasizing metabolic processes. Finally, through single-sample analysis, we identified a set of miRNAs induced by lunar orbital stressors that potentially target DNA methylation regulatory factors. The findings of this study broaden the understanding of space biological effects and lay a foundation for further exploration of the mechanisms by which deep space flight impacts plants.

## 1 Introduction

The space environment is characterized by strong radiation and microgravity, with radiation being a major limiting factor for human space exploration (Buchheim et al., 2019; Afshinnekoo et al., 2020). However, deep space exploration missions to celestial bodies such as the Moon and Mars face heightened exposure to galactic cosmic rays (GCR) and solar particle events (SPE) due to the absence of Earth’s magnetosphere protection (Chancellor et al., 2014; Afshinnekoo et al., 2020). This results in life forms on board being exposed to elevated radiation environments. Earlier studies have confirmed that the average radiation exposure on the lunar surface (1369 μSv/day) exceeds that encountered aboard the International Space Station (ISS) (731 μSv/day) (Zhang S et al., 2020). Therefore, it is essential to understand and evaluate the biological effects of high levels of cosmic radiation exposure in deep space for future deep space exploration missions.

Spaceflight, as a unique form of non-biological stress, induces changes in plants at the transcriptional and epigenetic levels. Near-Earth spaceflight has been observed to induce alterations in gene expression within various plant species including single cells of the fern Ceratopteris richardii, Arabidopsis thaliana, Mizuna, and Eruca sativa (Salmi and Roux, 2008; Sugimoto et al., 2014; Chandler et al., 2020; Kruse et al., 2020). These changes in gene expression result in modifications to protein expression in pathways associated with oxidative stress, DNA repair, response to stimulus, energy metabolism, amino acid metabolism, and protein metabolism (Sugimoto et al., 2014; Choi et al., 2019; Chandler et al., 2020; Kruse et al., 2020; Zeng et al., 2020). Moreover, modifications in chromatin structure and gene expression mediated by methylation are also influenced, suggesting that adaptation to spaceflight may occur through epigenetic changes (Casey et al., 2015). Zhou et al. revealed differential DNA methylation in Arabidopsis grown on the ISS, particularly at CHG and CHH sites. Additionally, 46% of the differentially methylated genes were associated with oxidative stress (Zhou et al., 2019). The alterations in DNA methylation levels induced by spaceflight can be transmitted to offspring (Ou et al., 2009; Xu et al., 2021).

DNA methylation involves the addition of a methyl group to the fifth carbon of cytosine residues, resulting in the formation of 5-methylcytosine (5mC) (Duan et al., 2018). This enzymatic process is mediated by DNA methyltransferases and utilizing S-adenosyl methionine (SAM) as the methyl donor (Law and Jacobsen, 2010; Duan et al., 2018). In plants, DNA methylation is classified into maintenance and *de novo* methylation based on their structural and functional attributes (Fan et al., 2022). The maintenance of methylation at symmetric CG, CHG, and asymmetric CHH sites is managed by distinct DNA methyltransferases (Cokus et al., 2008; Law and Jacobsen, 2010). Specifically, methyltransferase 1 (MET1), chromomethylase 3 (CMT3), and chromomethylase 2 (CMT2) are responsible for maintaining methylation at the CG, CHG, and CHH sites, respectively (Fan et al., 2022). *De novo* methylation is typically associated with gene silencing and plays a crucial role in mediating plant responses to abiotic stimuli (Kinoshita and Seki, 2014). *De novo* methylation occurs via canonical and non-canonical RNA-directed DNA methylation (RdDM) pathway. This process depends on 24 nt small interfering RNA (siRNA) and DOMAINS REARRANGED METHYLTRANSFERASE 2 (DRM2), forming a plant-specific DNA methylation mechanism (Zhang et al., 2018). It predominantly targets CHH sites and often initiates at regions of histone H3 lysine 9 methylation (H3K9me), leading to heterochromatin formation (Volpe et al., 2002). Moreover, SHH1, CLSY, and RNA polymerase IV (POL IV) proteins are recruited to these regions along with RDR2 and DCL3, resulting in the production of 24 nt siRNAs (Law et al., 2013; Erdmann and Picard, 2020). Furthermore, the non-canonical RdDM pathway involves the collaboration of POL II and RDR6 to produce 21-22 nt siRNAs similar to 24 nt siRNAs (Wu et al., 2012; Nuthikattu et al., 2013; Mccue et al., 2014). These siRNAs are subsequently loaded onto the Argonaute 4/6 protein (AGO4/6), where they pair with scaffold RNAs produced by POL V (Zhang et al., 2018). AGO4 interacts with DRM2 to catalyze *de novo* methylation with the assistance of other proteins (Cuerda-Gil and Slotkin, 2016). DNA demethylation involves both passive and active mechanisms (Li et al., 2017; Liu and Lang, 2019). In passive demethylation, 5mC is removed during DNA replication. Active DNA demethylation refers to the process by which methyl groups are actively removed from DNA through enzymatic mechanisms, typically involving DNA demethylases (Zhu, 2009; Fan et al., 2022).

DNA methyltransferases and proteins involved in RdDM pathways are notably implicated in the modulation of DNA methylation levels and are considered key determinants of such alterations (Ou et al., 2009). Here, we designated this group of enzymes and proteins as DNA methylation regulatory factors (MRs). Previous studies have indicated that microRNAs (miRNAs) are significant regulators of the DNA methylation process. They exert their influence by targeting transcripts and modulating the activities of proteins involved in DNA methylation, thereby instigating changes in methylation patterns (Huang et al., 2022; Saviana et al., 2023). In plants, miRNAs, typically 21-24 nucleotides long, are endogenous non-coding RNAs characterized by their hairpin structures (Tiwari et al., 2020). They commonly form complexes with the AGO1 protein to establish the RNA-induced silencing complex (RISC), which orchestrates the downregulation of target mRNA expression through cleavage (Chellappan et al., 2010; Wu et al., 2010). Recent investigations have highlighted the ability of miRNAs to impact DNA methylation levels by targeting MRs. For instance, miR820.1 has been identified to target *DRM2*, thereby modulating its expression levels and consequently regulating RdDM locus methylation levels (Park et al., 2022). Although there is some existing research, the mechanisms by which miRNAs regulate DNA methylation in spaceflight remain largely unexplored. Hence, we intend to initiate investigations in this domain to elucidate the intricate epigenetic regulatory pathways governing plants in lunar orbit flight.

On November 24, 2020, we exposed dry seeds of Japonica rice and thermoluminescent dosimeter (TLD) detection materials to 23 days of space radiation aboard the Chang’e-5 lunar probe. Upon their return to Earth, the samples were found to have received a cumulative dose of 59.85 mGy. Twenty days after their return, the seeds were germinated and cultivated under controlled conditions in an artificial climate chamber, allowing them to complete their entire life cycle. At both the tillering and heading stages, three plants were chosen randomly, and leaf samples were collected for transcriptome and DNA methylation sequencing analysis (n=3). Furthermore, due to the unique space environment, we employed single sample analysis methods to examine different rice plants from the spaceflight and ground control groups. Specifically, we first identified differential methylation, differentially expressed genes (DEGs) and miRNAs (DEmiRs). Subsequently, we constructed two single sample network (SSNs) for each sample at both the mRNA and miRNA levels. By comparing the topological features of each SSN between the spaceflight group and the ground control group, we identified differentially interacting genes (DIGs) and miRNAs (DImiRs). Moreover, we meticulously screened for differentially expressed and interacting MRs (DEMRs and DIMRs). Using psRNATarget online software, we predicted the DEmiRs and DImiRs targeting these MRs. In our study, we successfully analyzed the influence of lunar orbital flight stressors on rice DNA methylation.

## 2 Materials and methods

### 2.1 Plant materials

Japonica rice was selected as the plant material for this investigation. Dry seeds of japonica rice were transported aboard the Chang’e-5 lunar probe, enduring a 23-day spaceflight during which they accumulated a radiation dose of approximately 59.85 mGy. Twenty days later, 100 japonica rice seeds from both the ground control group and the spaceflight group were cultivated for their entire life cycle in an artificial climate chamber at Dalian Maritime University. We placed the rice seeds from each group into beakers and soaked them in deionized water. The seeds were then incubated in the artificial climate chamber at 25°C in the dark for four days to promote germination. During this period, we performed water changes twice daily, ensuring that the water was pre-warmed in the artificial climate chamber prior to use. After germination, we adjusted the chamber temperature to 28°C/25°C (day/night) and controlled the photoperiod to 14 hours of light and 10 hours of darkness, with a light intensity set at 20,000 lux. Additionally, we utilized Yoshida culture solution at pH 5.5 for the liquid culture of the rice plants.

### 2.2 Genomic DNA and total RNA extraction

Three rice plants were randomly selected from both the ground control group and the spaceflight group, designated as the ground control group: C1, C2, C3; and the spaceflight group: S1, S2, S3. Leaf samples were collected at the tillering and heading stages and stored at -80°C, designated as the tillering stage ground control group (TC): TC1, TC2, TC3; tillering stage spaceflight group (TS): TS1, TS2, TS3; heading stage ground control group (HC): HC1, HC2, HC3; and heading stage spaceflight group (HS): HS1, HS2, HS3.

Genomic DNA was extracted from rice leafs using the Universal Genomic DNA Extraction Kit (TaKaRa, Dalian, China). DNA concentration was measured. Total RNA was isolated and purified from rice leaves using TRIzol reagent (Invitrogen, Carlsbad, CA, USA) according to the manufacturer’s protocol. The quantity and purity of RNA from each sample were quantified using NanoDrop ND-1000 spectrophotometer (NanoDrop, Wilmington, DE, USA). The RNA integrity was precisely assessed using an Agilent Bioanalyzer 2100. The RNA concentration of each sample > 100 ng/µl, RNA integrity number > 6.0, OD260/280 > 1.8 and total RNA > 20µg.

### 2.3 Whole-genome bisulfite sequencing library construction

To construct whole-genome bisulfite sequencing library, genomic DNA was randomly fragmented to 200-300 bp using a Covaris S220. Subsequently, bisulfite treatment was performed using the Accel-NGS^®^ Methyl-Seq DNA Library Kit (Swift Biosciences, Beijing, China). During this process, unmethylated cytosines (C) were converted to uracil (U), which were further converted to thymine (T) after PCR amplification, while methylated cytosines remained unchanged. Finally, PCR amplification was conducted to add adapters, resulting in the final DNA library. The library concentration was quantified by Qubit2.0 Flurometer (Life Technologies, CA, USA) and the results were quantified via quantitative PCR, and the insert size was assayed on Agilent Bioanalyzer 2100 system. The library preparations were sequenced on an Illumina HiSeq/NovaSeq platform.

### 2.4 RNA-seq library construction

We entrusted Novogene (Beijing, China) to construct strand-specific libraries using a method that removes ribosomal RNA (Parkhomchuk et al., 2009). First, ribosomal RNA was depleted from total RNA. Subsequently, the RNA was fragmented into short fragments of 250-300 bp using RNase R enzyme. The fragmented RNA served as a template, and random oligonucleotides as primers were used to synthesize the first strand of cDNA. Subsequently, RNase H was employed to degrade the RNA strand, enabling the synthesis of the second strand of cDNA by DNA polymerase I using dNTPs (dUTP, dATP, dGTP, and dCTP) as substrates. The purified double-stranded cDNA underwent end repair, A-tailing, and adapter ligation, followed by size selection of cDNA fragments around 350-400 bp using AMPure XP beads. The cDNA second strand containing uracil was degraded using USER enzyme. Finally, PCR amplification was performed to obtain the library. The library concentration was quantified by Qubit2.0 Flurometer (Life Technologies, CA, USA) and the results were quantified via quantitative PCR, and the insert size was assayed on Agilent Bioanalyzer 2100 system. The library preparations were sequenced on an Illumina PE150 platform.

The library construction was carried out using the Small RNA Sample Pre Kit (Novogene, Beijing, China). Utilizing the unique structural features of small RNAs (a complete phosphate group at the 5’ end and a hydroxyl group at the 3’ end), total RNA was used as the starting material. The small RNA ends were directly ligated with adapters, followed by reverse transcription to synthesize cDNA. Subsequently, PCR amplification was performed, and the target DNA fragments were separated by PAGE gel electrophoresis. The recovered DNA fragments from gel excision constituted the cDNA library. The library concentration was quantified by Qubit2.0 Flurometer (Life Technologies, CA, USA) and the results were quantified via quantitative PCR, and the insert size was assayed on Agilent Bioanalyzer 2100 system. The library preparations were sequenced on an Illumina SE50 platform.

### 2.5 BS-Seq data processing and analysis

Raw 150-nt paired-end reads were subjected to quality control (QC) filters using FASTQC (http://www.bioinformatics.babraham.ac.uk/projects/fastqc/) and trimmed using Trimmomatic v0.39. Bismark was utilized for alignment analysis of methylation data against the reference genome (MSU_v7.0). PCR-generated duplicate reads were removed from each methylome. The methylation level of individual cytosines was calculated by dividing the number of methylated cytosines by the total read depth: [mC/(mC + non-mC)]. Only sites with coverage of four or more were considered.

### 2.6 mRNA-Seq data processing

The raw data underwent filtering, sequencing error rate assessment, and examination of GC content distribution to obtain clean reads. Hisat2 software (http://ccb.jhu.edu/software/hisat2) was employed to align RNA-Seq sequencing data to the rice reference genome (MSU_v7.0) (Kim et al., 2015; Vieth et al., 2019). StringTie, utilizing a network flow algorithm, was used to assemble and quantify transcripts and genes from uniquely mapped reads on the genome (Pertea et al., 2015). Gene expression levels in RNA-seq were represented as the number of fragments per kilobase of transcript per million mapped fragments (FPKM) for each gene (**Supplementary Table S1**).

### 2.7 sRNA-Seq data processing

The raw data underwent filtering to obtain clean reads, followed by assessment of sequencing error rates and GC content distribution. Subsequently, small RNAs within the length range of 18-30 nucleotides were selected for further analysis. The length-filtered small RNAs were aligned to the reference sequences using Bowtie (Langmead et al., 2009). The reads mapped to the reference sequences were compared with specified range sequences in miRBase to obtain detailed information on matched small RNAs for each sample. Expression levels of miRNAs in each sample were quantified, and normalization of expression levels was performed using TPM normalization (**Supplementary Table S2**).

### 2.8 DEGs, DEmiRs, and differential methylation analysis

We first analyzed the differences in methylation and transcriptome profiles between the spaceflight group and the ground control group (n=3). Differential methylation analysis was performed utilizing DSS software to detect divergent methylated regions (DMRs), with statistical significance determined at a threshold of P-values< 0.05. We defined genes with DMRs in their promoter and gene body as differentially methylated genes (DMGs). Differentially expressed genes (DEGs) were determined using DESeq2 with a twofold change and P-values< 0.05. Additionally, we designated the genes involved in DNA methyltransferases and RdDM as DNA methylation regulatory factors (MRs), with differentially expressed genes referred to as DEMRs (P-values< 0.05). Differentially expressed miRNAs (DEmiRs) also were determined using DESeq2 with a twofold change and P-values< 0.05.

We further analyzed the methylation and transcriptome profiles of individual rice plants subjected to lunar induction using single sample analysis methods. Differential methylation analysis was performed utilizing DSS software to detect DMRs, with statistical significance determined at a threshold of P-values< 0.05. Moreover, the mRNAs and miRNAs exhibiting zero expression levels were removed. Subsequently, compared to the ground control group, we obtained differential changes in the FPKM values of genes at the tillering and heading stages for three different rice individuals in the spaceflight group. DEGs were identified using single sample t-test with a twofold change and P-values< 0.05. DEmiRs and DEMRs were identified using a significance threshold of P-values< 0.05.And the single sample t-tests were conducted using the ttest_1samp () function from the scipy Python package (Li et al., 2023).

### 2.9 Differentially interacting genes (DIGs) miRNAs (DImiRs) analysis

We utilized single-sample networks (SSN) to identify contrasting patterns of gene interactions. According to the methods provided in reference (Kuijjer et al., 2019; Zhang et al., 2024b), the SSNs were constructed for 12 samples using LIONESS respectively (an mRNA-SSN and a miRNA-SSN were constructed for each sample separately).

To ascertain the differential interaction patterns between genes/miRNAs in the ground control and spaceflight groups, we extracted degrees as node features in the SSNs. The degree refers to the number of neighbors for each node.

Subsequently, we selected the genes/miRNAs present in the SSN and constructed degree vectors d for each gene/miRNA across 12 samples (n=12) according to Equation 1. For any given molecule, $d_{i}$ denotes the degree in the i -th SSN (if this molecule is not present in the i -th SSN, then $d_{i}$ =0).


(1) $$d=(d_{1},\;d_{2},\;\cdots \;,\;d_{n})$$

Finally, we conducted the single-sample t-tests on the $d_{GC\;}$ = ( $d_{1}$ , $\;d_{2}$ , $\;d_{3}$ ) (degree vector of a gene/miRNA in the ground control group) and $d_{SP\;\;}$ = ( $d_{4}$ ) (degree vector of a gene/miRNA in a spaceflight individual), defining molecules with P-values< 0.05 as DIGs, DImiRs. And the single-sample t-tests were conducted using the ttest_1samp () function from the scipy Python package (Li et al., 2023).

### 2.10 Prediction of miRNAs Targeting MRs

To identify miRNAs targeting MRs, we used the psRNATarget online software (2017 Update) (zhaolab.org/psRNATarget/analysis) with default parameters for prediction. The miRNA and mRNA sequences for target prediction are shown in **Supplementary Table S3**, and all miRNAs and corresponding predicted targets are also provided in **Supplementary Table S3**.

### 2.11 Gene Ontology(GO) enrichment analysis

DEGs, DIGs, and DMGs were subjected to GO enrichment analysis using the Gene Ontology Resource (GeneOntology.org), with statistical significance established at P-values< 0.05. To assess comparative biological functions, we defined the significance of enrichment as -log10 (P-values).

### 2.12 Validation of DEGs and DEmiRs

The materials used for the qPCR experiments are identical to those utilized for methylation and transcriptome sequencing. In this study, qRT-PCR technology was employed to validate mRNA expression levels, with OsActin serving as the reference gene. Concurrently, the stem-loop RT-PCR technique was utilized to verify miRNA expression levels, with U6 serving as the reference gene. The relative expression levels of miRNAs and mRNAs were calculated using the Ct (2^-ΔΔCt^) method. To ensure the reliability of the experimental results, all PCR reactions were performed in triplicate technically, and each reaction in the ground control group was subjected to three biological replicates. The primers used in the experiments are listed in **Supplementary Table S4**.

## 3 Results

### 3.1 Features of rice DNA methylome after lunar orbital flight

**Figure 1:**
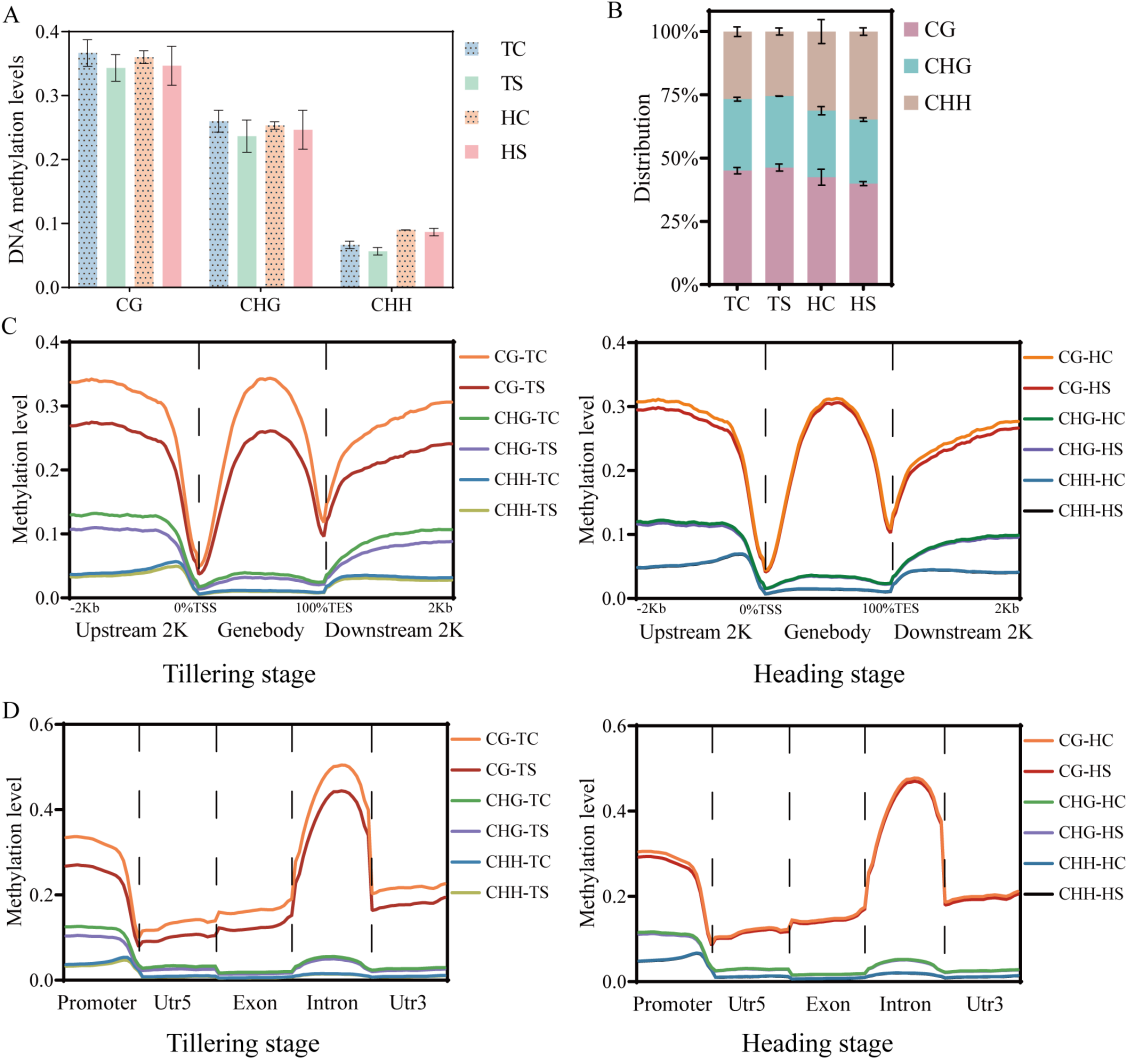
DNA Methylation characteristics during Tillering and Heading Stages of Rice. **(A)**. The average DNA methylation levels of CG, CHG, and CHH methylation in rice. *Shading represents ground control group.* **(B)**. The average frequencies (%) of CG, CHG, and CHH methylation in rice. **(C)**. DNA methylation profiles of mCG, mCHG and mCHH surrounding genes (within 2kb) in rice. **(D)**. DNA methylation profiles of mCG, mCHG, and mCHH in gene functional regions of rice. TC, Ground control group during the tillering stage; TS, Spaceflight group during the tillering stage; HC, Ground control group during the heading stage; HS, Spaceflight group during the heading stage. The data (mean ± SD) are the means of three replicates with standard errors shown by vertical bars, n = 3. T-test.

Whole-genome analysis by different methylation contexts revealed that, compared to the ground control group, the average methylation levels of CG, CHG, and CHH showed a reduction in the spaceflight group during the tillering and heading stages (P-value > 0.05) (**Figure 1A**). During the tillering stage, the average frequency of different contexes of methylation was CG > CHG > CHH, whereas at the heading stage, the average frequency was CG > CHH > CHG (**Figure 1B**). During the tillering stage, spaceflight resulted in a reduction in CG and CHG methylation levels within the 2K upstream and downstream regions and functional regions of genes. This phenomenon was less pronounced during the heading stage (**Figures 1C, D**).

### 3.2 Characterization of differentially methylated regions (DMRs) induced by lunar orbit stressors

**Figure 2:**
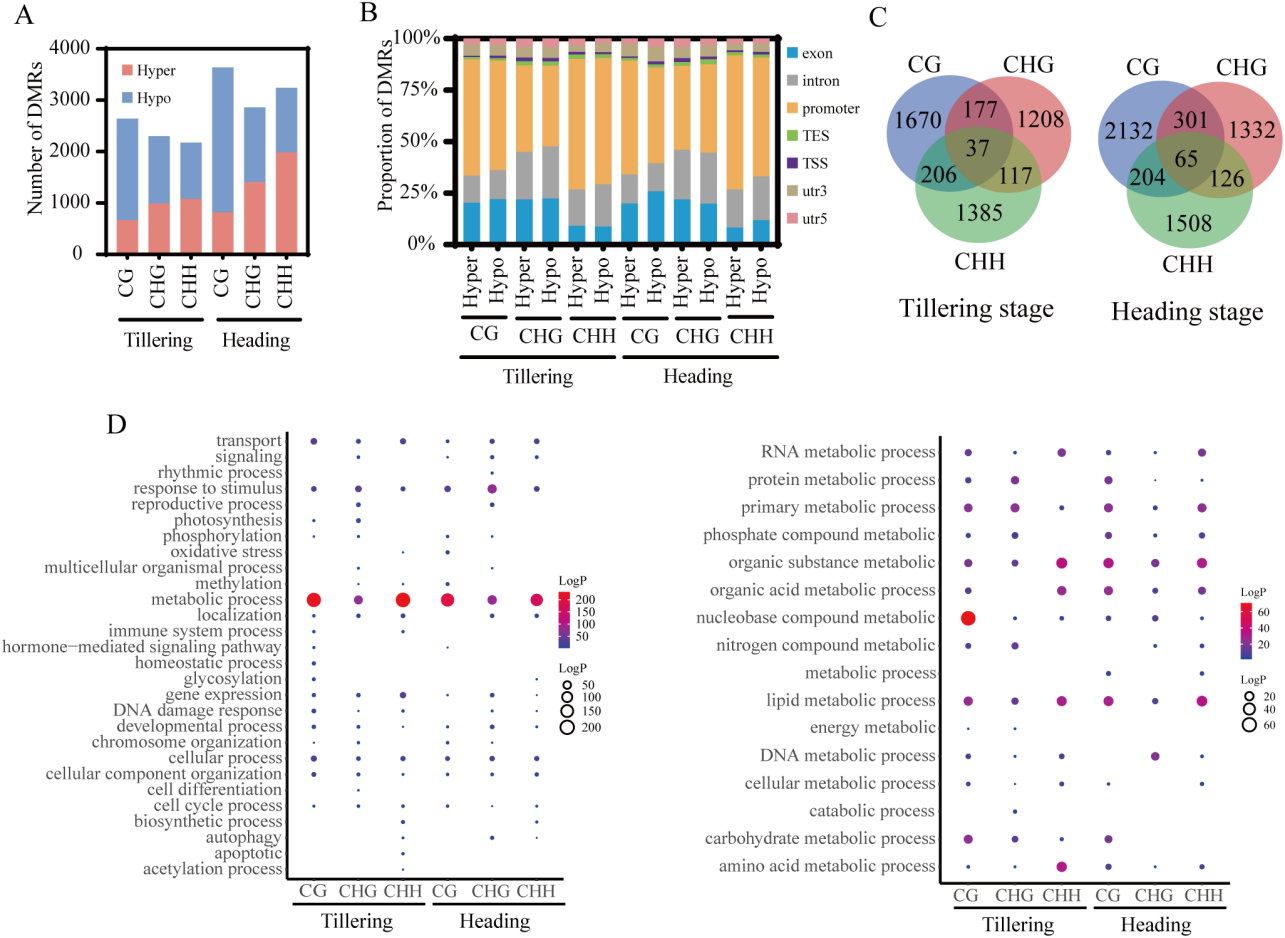
Characterization of DMRs in different cytosine contexts. **(A)** Number of DMRs near to protein coding genes in rice. **(B)** Genomic compositions of DMRs surrounding genes. **(C)** Overlap of DMGs with different methylation contexts. **(D)** GO analysis of DMGs (P-value< 0.05). The left figure displays GO enrichment analysis of DMGs (P-value< 0.05), while the right figure illustrates GO enrichment analysis of metabolic processes among DMGs (P-value< 0.05).

To investigate changes in methylation near genes, we focused on DMRs in promoters and gene bodies of all functional genes. Compared to the tillering stage, more DMRs were identified in the heading stage samples. Additionally, except for CHH-DMRs in the heading stage samples, the number of DMRs with decreased methylation levels was greater than those with increased methylation levels (**Figure 2A**). Approximately 50% of CG-DMRs, CHG-DMRs, and CHH-DMRs were located in promoter regions (**Figure 2B**).

Over 73% of DMGs in both tillering and heading stage samples were exclusively associated with one context of DMR (**Figure 2C**). To comprehend the biological functions of different contexts of DMGs, GO enrichment analysis was conducted (**Figure 2D**). During the tillering and heading stages, CG-DMGs, CHG-DMGs, and CHH-DMGs were primarily associated with metabolic processes, response to stimulus, and transport processes. Metabolic processes included nucleic acid (DNA, RNA) metabolism, lipid metabolism, and organic substance metabolism, among others.

### 3.3 The association analysis of DNA methylome and transcriptome

**Figure 3:**
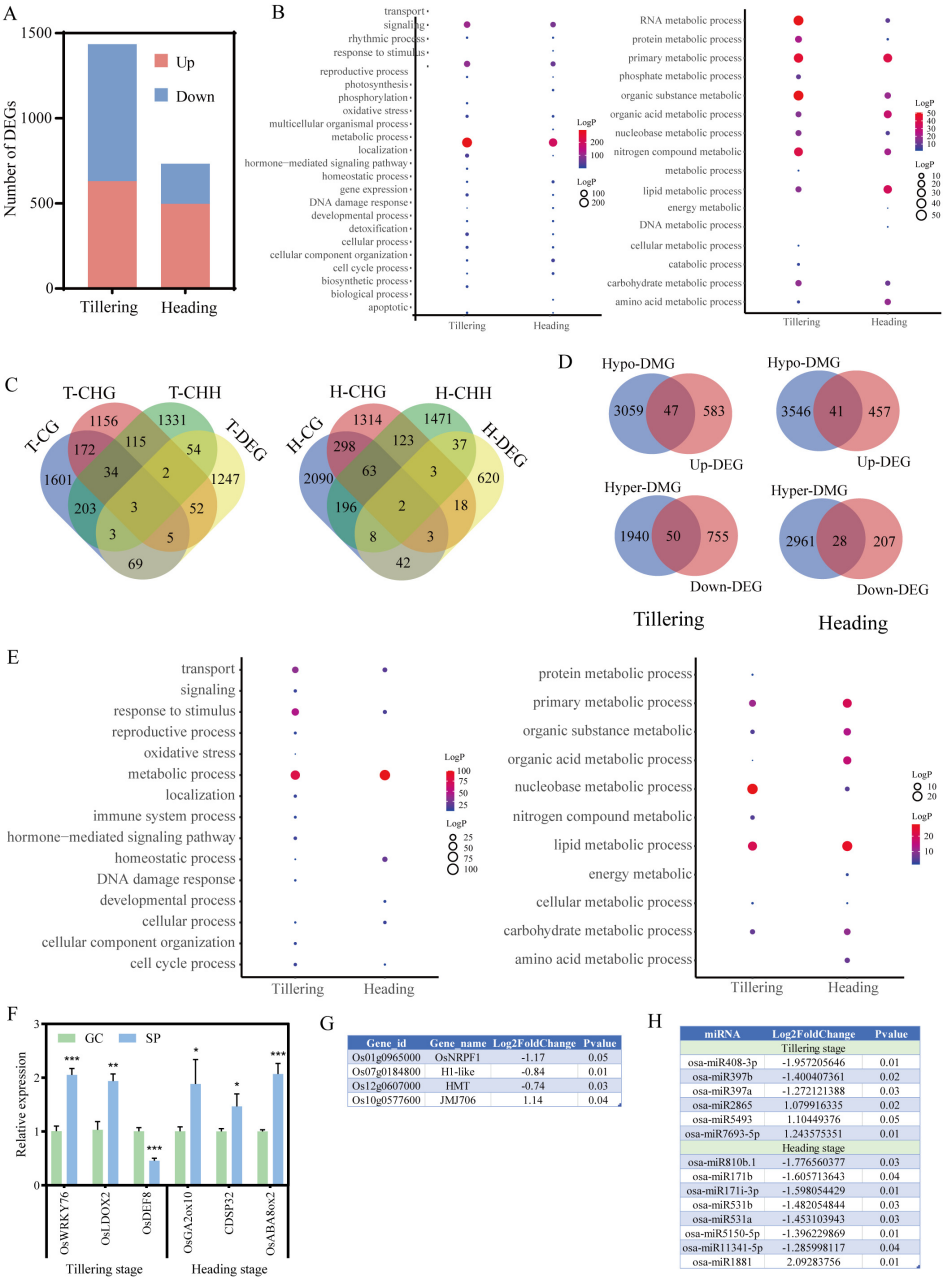
The association analysis of DNA methylome and RNA transcriptome. **(A)** Number of DEGs in rice. **(B)** GO analysis of DEGs from different methylation contexts (P-value< 0.05). The left figure displays GO enrichment analysis of DEGs (P-value< 0.05), while the right figure illustrates GO enrichment analysis of metabolic processes among DEGs (P-value< 0.05). **(C)** The Venn figure illustrates the association between three contexts of DMGs and spaceflight induced DEGs during the tillering and heading stages of rice. **(D)** The Venn figure illustrates the association between methylation levels and spaceflight-induced DEG during the tillering and heading stages of rice. **(E)** GO analysis of overlapping genes between DMGs and DEGs (P-value< 0.05). The left figure displays GO enrichment analysis of overlapping genes between DMGs and DEGs (P-value< 0.05), while the right figure illustrates GO enrichment analysis of metabolic processes among overlapping genes between DMGs and DEGs (P-value< 0.05). **(F)** Validation of Gene Expression Levels by qRT-PCR. **(G)** Transcript level of DEMRs. **(H)** Transcript level of DEmiRs. Ground control group (GC) and Spaceflight group (SP) data (mean ± SD) are the means of three replicates with standard errors shown by vertical bars, n = 3; “*” represents P-value<0.05, “**” represents P-value<0.01 and “***” represents P-value<0.001. T test.

This study employed the same material for both methylation analysis and RNA-Seq sequencing, with each sample obtaining three replicates simultaneously. By comparing the RNA-Seq data of ground control and spaceflight groups, 1435 and 733 DEGs were identified in the tillering and heading stage samples, respectively (|log2FC| > 1, P-values< 0.05) (**Figure 3A**). GO enrichment analysis (**Figure 3B**) reveals that DEGs in both growth stages are primarily involved in metabolic processes, response to stimulus, and transport processes. This is consistent with the functions associated with DMGs. We selected three stress-related genes that may have their expression altered due to DNA methylation during the tillering and booting stages for RT-qPCR validation (**Figure 3F**). The results indicated that the differential gene expression caused by the lunar orbital stressors underwent significant changes.

To explore the relationship between methylation alterations and transcriptional modifications, a correlation analysis was conducted between DEGs and DMGs. It was found that 188 and 113 DMGs overlapped with DEGs in the tillering and heading stage samples, respectively. Notably, over 85% of DEGs overlapp with only one context of DMG (**Figure 3C**). We also observed that in the tillering and heading stages, 47 and 41 hypomethylated DMGs overlapped with upregulated DEGs, respectively, while 50 and 28 hypermethylated DMGs overlapped with downregulated DEGs (**Figure 3D**). Although only a small subset of spaceflight-induced genes undergo differential methylation, these genes are primarily involved in regulatory processes such as primary metabolic, nucleobase metabolic, and lipid metabolic pathways (**Figure 3E**).

### 3.4 Impact of lunar orbital flight on miRNA and DNA methylation regulators in rice

We further analyzed the impact of lunar orbital flight on DNA methylation regulators. Initially, we compiled a list of MRs through a literature review (**Supplementary Table S5**) (Chen and Zhou, 2013; Bradbury et al., 2014; Sun et al., 2021; Huang et al., 2022; Wang et al., 2022; Yan et al., 2022; Lee et al., 2023). The subsequent step involved selecting MRs from the aforementioned DEGs to obtain DEMRs. We found that during the tillering stage, the expression levels of *H1-like, HMT, JMJ706, and OsNRPF1* were altered, with only *JMJ706* being upregulated (**Figure 3G**).

Next, we analyzed the impact of lunar orbital flight on miRNAs and identified 6 DEmiRs (3 upregulated, 3 downregulated) during the tillering stage and 8 DEmiRs (1 upregulated, 7 downregulated) during the heading stage (**Figure 3H**). The target genes of these DEmiRs did not overlap with DEMRs (**Supplementary Table S6**).

### 3.5 The single sample analysis of DNA methylation profiles in different rice individuals

**Figure 4:**
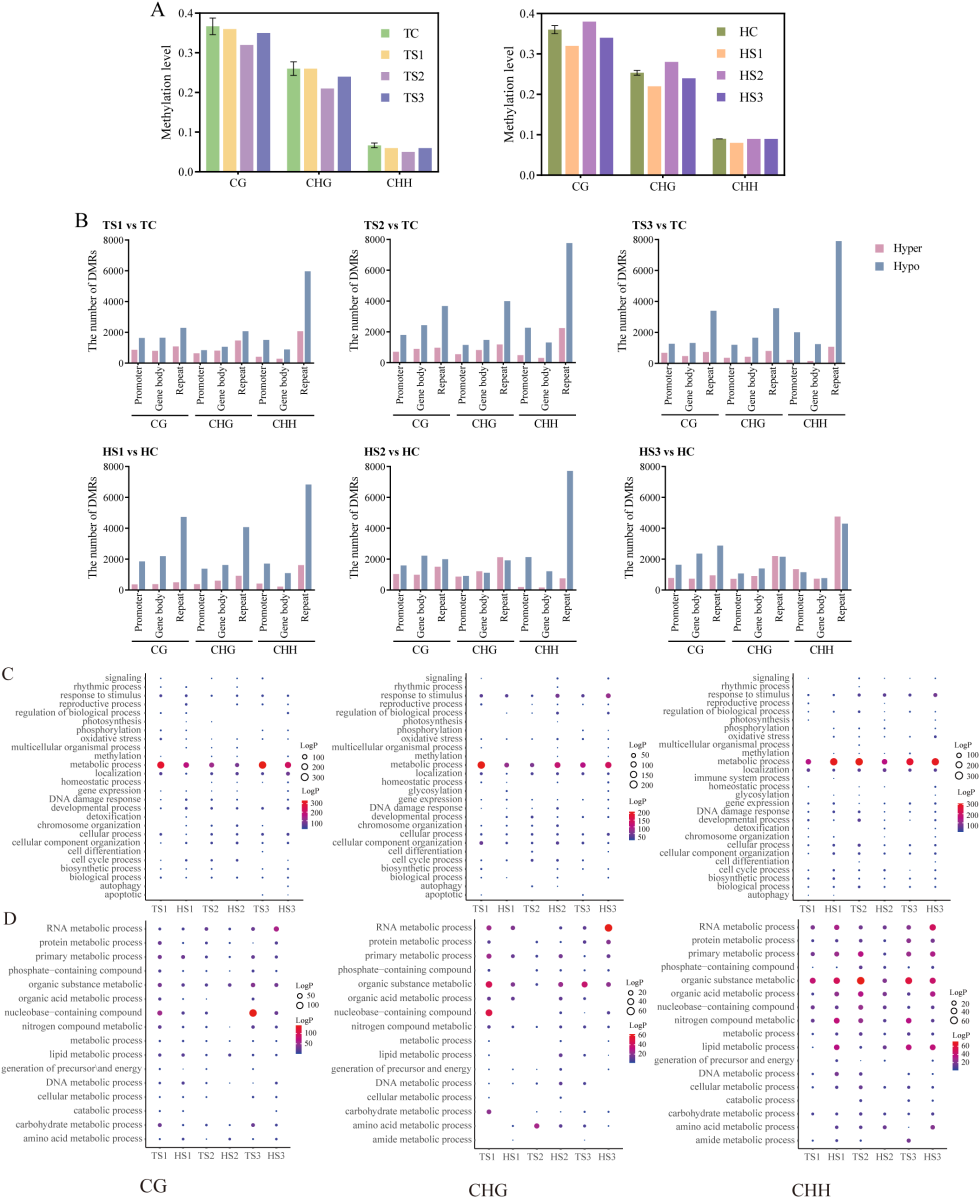
DNA methylation characteristics during tillering and heading stages of each rice sample. **(A)** Average DNA methylation levels of CG, CHG, and CHH methylation in each rice sample. **(B)** Number of DMRs in each rice sample. **(C)** GO analysis of DMGs of CG, CHG, and CHH contexts (P-value< 0.05). **(D)** GO enrichment analysis of metabolic processes among DMGs of CG, CHG, and CHH contexts (P-value< 0.05). TC, Tillering stage ground control group; TS1,TS2,TS3, Tillering stage spaceflight group; HC, Heading stage ground control group; HS1,HS2,HS3, Heading stage spaceflight group. Ground control group data (mean ± SD) are the means of three replicates with standard errors shown by vertical bars, n = 3; Spaceflight group data analyzed as individual samples, n=1. The single sample T test.

We conducted an individual analysis of the 12 rice samples used for methylation and transcriptome sequencing in the two growth stages. According to the whole-genome analysis of different methylation contexts, lunar orbital flight induced inconsistent changes in individual rice plants. Overall, the average methylation levels of CG, CHG, and CHH contexts were slightly reduced in the spaceflight samples compared to the ground control group, with the exception of HS2, though not statistically significant (**Figure 4A**). Similar to inter-group analysis (n=3), during the tillering stage, spaceflight resulted in a reduction in CG and CHG methylation levels within gene the 2K upstream and downstream regions and functional regions. This phenomenon was less pronounced during the heading stage (**Supplementary Figures S1A,B**). Spaceflight induces the generation of DMRs in rice samples during both the tillering and heading stages, with a greater abundance of hypomethylated DMRs than hypermethylated DMRs located within the promoter regions (**Figure 4B**; **Supplementary Table S7**).

GO enrichment analysis of DMGs to CG, CHG, and CHH contexts in the genome (**Figures 4C, D**) reveals a significant focus on biological processes such as metabolism, localization, cell cycle regulation, DNA damage and repair, and response to stimulus across different rice individuals. DMGs implicated in metabolic functions across diverse individuals are notably concentrated in processes associated with RNA, protein, organic substance, primary, and nitrogen metabolism. Particularly noteworthy is the pronounced enrichment of metabolic processes among DMGs linked to CHH sites (P-values<0.05).

### 3.6 The single sample analysis of transcriptomic changes in different rice individuals

**Figure 5:**
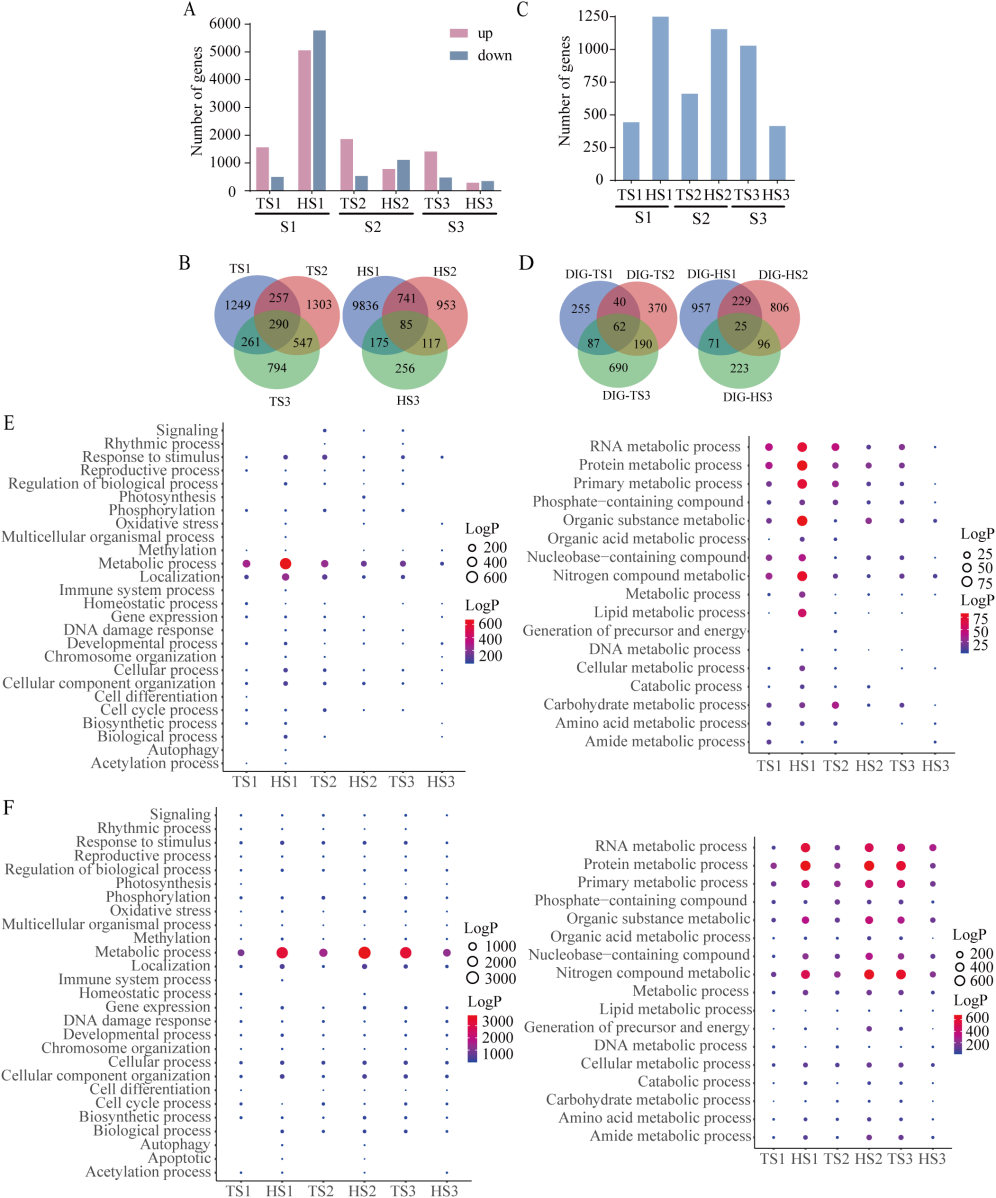
Transcriptomic changes during tillering and heading stages of each rice sample. **(A)** Number of DEGs in each rice sample. **(B)** The Venn diagram illustrates the overlap of DEGs among three samples. **(C)** Number of DIGs in each rice sample. **(D)** The Venn diagram illustrates the overlap of DIGs among three samples. **(E)** GO analysis of DEGs (P-value< 0.05). The left figure displays GO enrichment analysis of DEGs (P-value< 0.05), while the right figure illustrates GO enrichment analysis of metabolic processes among DEGs (P-value< 0.05). **(F)** GO analysis of DIGs (P-value< 0.05). The left figure displays GO enrichment analysis of DIGs (P-value< 0.05), while the right figure illustrates GO enrichment analysis of metabolic processes among DIGs (P-value< 0.05). TS1, TS2,TS3, Tillering stage spaceflight group; HS1, HS2, HS3, Heading stage spaceflight group. Spaceflight group data analyzed as individual samples, n=1. The single sample T test.

Through transcriptome analysis of each rice sample during the two growth stages, we found that, compared to ground control group, the number of upregulated differentially expressed genes in each sample (TS1, TS2, TS3) during the tillering stage was approximately three times higher than that of downregulated genes. In contrast, during the heading stage, each sample (HS1, HS2, HS3) exhibited more downregulated differentially expressed genes than upregulated genes (**Figure 5A**). Additionally, there was a relatively small overlap in DEGs between the tillering and heading stages, indicating that gene changes at different developmental stages are unique to each individual (**Supplementary Figure S2**). Specifically, there were 290 and 85 common DEGs during the tillering and heading stages, respectively (**Figure 5B**). These results suggest that the impact of spaceflight on plant gene expression is both stage specific and individual dependent, implying potential differences in adaptive responses to spaceflight between the tillering and heading stages. At the same time, we also found that the DEGs and DMGs of each rice sample overlapped, with over 80% of DEGs overlapping with only one context of DMG (**Supplementary Figure S3B**).

To comprehensively capture the information regarding functional gene changes, we further employed the SSN method to analyze gene interaction patterns. The results revealed that during the tillering and heading stages, there were 444 and 1282 DIGs for the S1 individual, 662 and 1156 DIGs for the S2 individual, and 1029 and 415 DIGs for the S3 individual, respectively (**Figure 5C**). From the varying number of DIGs across different rice individuals at two developmental stages, we observed a dynamic complementary phenomenon: individuals with relatively fewer changes during the tillering stage exhibited an increase in changes during the heading stage, while those with more changes during tillering showed a reduction in changes during heading. Upon scrutinizing the common DIGs among the three individuals during both the tillering and heading stages, we respectively found a mere 62 and 25 DIGs (**Figure 5D**). This outcome further confirms the heterogeneity in gene interaction patterns among different rice individuals.

The GO enrichment analysis of DEGs and DIGs revealed similar biological processes across all individuals, primarily focusing on metabolic processes (**Figures 5E, F**). Specifically, the enriched metabolic processes included RNA metabolism, protein metabolism, primary metabolism, and nitrogen metabolism, among others. DIGs reflect the initiation of oxidative stress response as early as the tillering stage, persisting into the heading. Furthermore, biological processes such as signaling, biological rhythm, photosynthesis, chromatin organization, cell differentiation, acetylation processes, and apoptosis were enriched among DIGs from S2 and S3 individuals. Additionally, these functions exhibit dynamic trends from the tillering to heading stages.

### 3.7 The single sample analysis of miRNA and DNA methylation regulatory factors in different rice individuals

**Figure 6:**
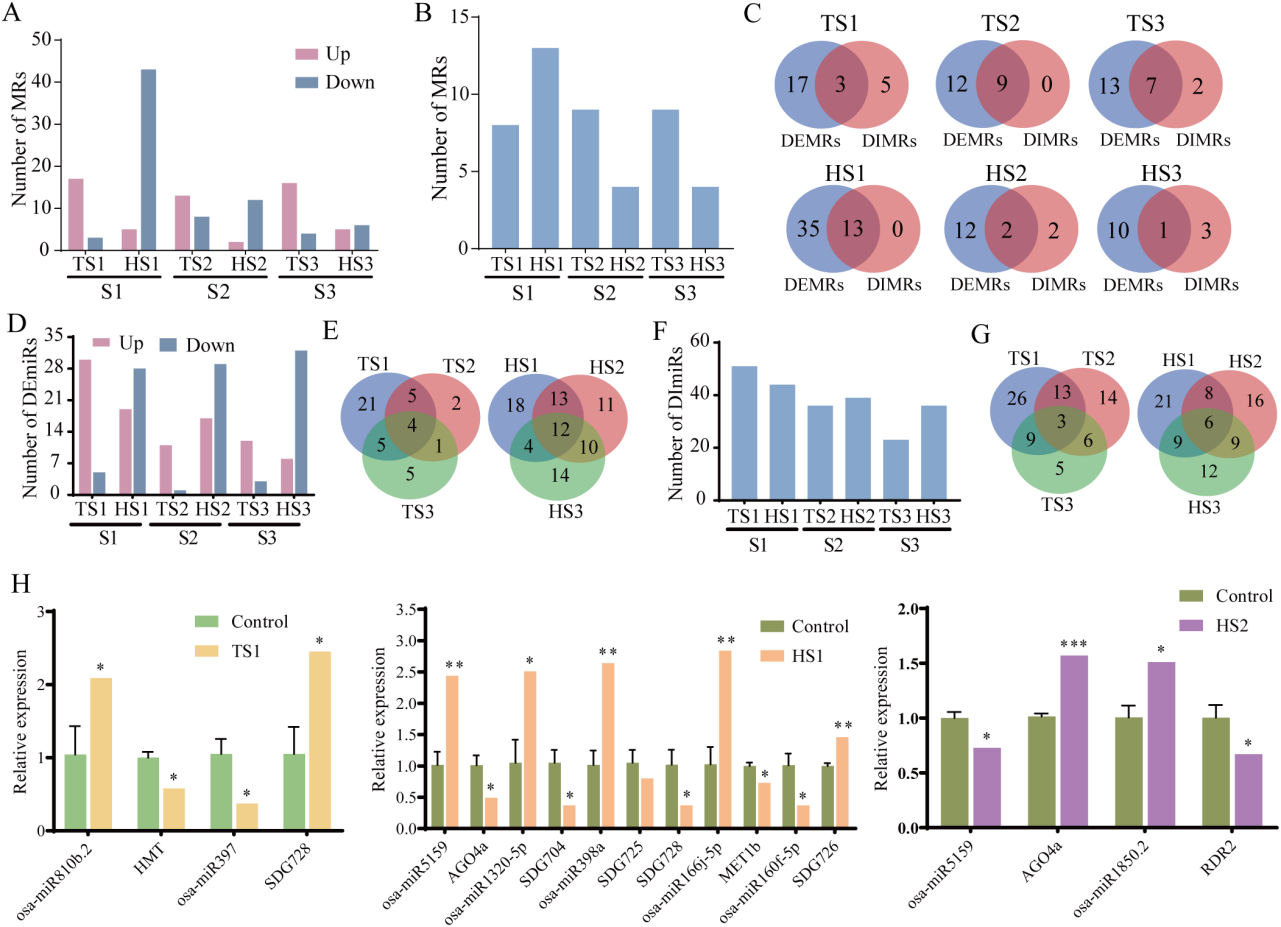
MRs and miRNAs analysis of different rice individuals. **(A)** The number of DEMRs. **(B)** The number of DIMRs. **(C)** The intersection of DEMRs and DIMRs among different rice individuals at the tillering and heading stages. **(D)** The number of DEmiRs. **(E)** The intersection of DEmiRs among different rice individuals at the tillering and heading stages. **(F)** The number of DImiRs. **(G)** The intersection of DImiRs among different rice individuals at the tillering and heading stages. **(H)** The expression levels of selected DEmiRs and DEMRs. Ground control group data (mean ± SD) are the means of three replicates with standard errors shown by vertical bars, n = 3; Spaceflight group data analyzed as individual samples, n=1. “*” represents P-value< 0.05, “**” represents P-value< 0.01 and “***” represents P-value< 0.001. The single sample T test.

We further analyzed the effects of spaceflight on the expression and interaction patterns of MRs across different rice individuals. The results revealed that at the tillering stage, most MRs were upregulated, whereas at the heading stage, the majority were downregulated (**Figure 6A**; **Supplementary Table S8**). At the gene interaction level, we found that during the tillering and heading stages, the S1 individual respectively exhibited 8 and 13 DIMRs, while the S2 individual respectively had 9 and 4, and the S3 individual had 9 and 4 DIMRs (**Figure 6B**; **Supplementary Table S9**). Additionally, there was an intersection between DEMRs and DIMRs, indicating concurrent responses of gene expression and interactions to stressors (**Figure 6C**).

Next, we analyzed the expression and interactions of miRNAs in different rice individuals under lunar orbit stressors. Notably, during the tillering stage, the number of induced upregulated differential miRNAs surpassed the downregulated count, whereas the opposite trend was observed during the heading stage. Furthermore, a higher count of DEmiRs was observed during the heading stage (**Figure 6D**; **Supplementary Table S10**). At both the tillering and heading stages, there respectively were 4 and 12 DEmiRs common to three individuals, underscoring the individual-specific alterations in DEmiRs triggered by spaceflight (**Figure 6E**). During the tillering and heading stages, the S1 individual exhibited 51 and 44 DImiRs, while the S2 individual displayed 36 and 39 DIMs, and the S3 individual showed 23 and 36 DImiRs (**Figure 6F**; **Supplementary Table S11**). There were dynamic and complementary changes in the number of DImiRs observed among different individuals during these developmental stages. The shared DImiRs among all individuals during both tillering and heading stages respectively were 3 and 6 (**Figure 6G**), suggesting distinct alterations in miRNA interaction patterns among different individuals.

### 3.8 The single-sample analysis of miRNAs potentially targeting MRs in different rice individuals

We conducted miRNAs target prediction for MRs using psRNATarget online software. Our analysis revealed two categories of miRNAs (DEmiRs and DImiRs) corresponding to DEMRs and DIMRs (**Table 1**; **Supplementary Table S12**, **Supplementary Figures S4**, **S5**). Herein, we identified eight pairs of DEMRs and DEmiRs with upregulation and downregulation in opposite directions and validated their expression using qRT-PCR (**Figure 6H**). The qRT-PCR results confirmed significant differences in the expression of the above genes and miRNAs under spaceflight conditions. Moreover, employing the SSN method, we identified 11 DImiRs targeting with DIMRs. Notably, these two sets of differential miRNAs demonstrate an overlap. For example, osa-miR5159 targets *AGO4* as predicted.

**Table 1:** The key miRNAs and MRs.

Sample	Developmental Stage	Method	miRNAs	MRs	Gene name	Gene description	DNA methylation
S1	Tillering stage	Fold change	osa-miR397b^*^↓	Os05g0490700^*^↑	SDG728	Histone H3K9 methyltransferase	RdDM
			osa-miR810b.2^*^↑	Os12g0607000^**^↓	HMT	Homocysteine S-methyltransferase 2	RdDM
		SSN	osa-miR5083↑	Os09g0307800↓	SDG724	Histone methyltransferase	RdDM
	Heading stage	Fold change	osa-miR5159^**^↑	Os01g0275600^**^↓	AGO4a	Argonaute protein	RdDM
			osa-miR398a^**^↑	Os02g0554000^*^↓	SDG725	Histone H3K36 methyltransferase	RdDM
			osa-miR398a^**^↑	Os05g0490700^**^↓	SDG728	Histone H3K9 methyltransferase	RdDM
			osa-miR166j-5p^*^↑	Os07g0182900^*^↓	MET1b	methyltransferase	CG site maintains methylation
			osa-miR160f-5p^*^↓	Os07g0435900^**^↑	SDG726	Histone H3K9 methyltransferase	RdDM
			osa-miR1320-5p^*^↑	Os11g0602200^***^↓	SDG704	Histone methyltransferase	RdDM
		SSN	osa-miR160f-5p^*^↓	Os07g0435900^**^↑	SDG726	Histone H3K9 methyltransferase	RdDM
			osa-miR11339-5p↑	Os07g0435900^**^↑	SDG726	Histone H3K9 methyltransferase	RdDM
			osa-miR5159^**^↑	Os01g0275600^**^↓	AGO4a	Argonaute protein	RdDM
S2	Tillering stage	SSN	osa-miR5501↑	Os05g0445900^*^↑	ROS1c	DNA demethylase	DNA demethylation
			osa-miR5079a↑	Os10g0577600^**^↑	JMJ706	H3K9 demethylase	RdDM
			osa-miR169a↓	Os01g0527600^*^↓	RDR6	RNA-dependent RNA polymerase 6	RdDM
			osa-miR2863c↑	Os07g0182900^*^↑	MET1b	methyltransferase	CG site maintains methylation
	Heading stage	Fold change	osa-miR5159^*^↓	Os01g0275600^*^↑	AGO4a	Argonaute protein	RdDM
			osa-miR1850.2^*^↑	Os04g0465700^*^↓	RDR2	RNA-dependent RNA polymerase 2	RdDM
		SSN	osa-miR5159^*^↓	Os01g0275600^*^↑	AGO4a	Argonaute protein	RdDM
			osa-miR1874-5p↑	Os01g0275600^*^↑	AGO4a	Argonaute protein	RdDM
S3	Tillering stage	SSN	osa-miR169a↓	Os01g0527600↓	RDR6	RNA-dependent RNA polymerase 6	RdDM
			osa-miR2863c↓	Os07g0182900^**^↓	MET1b	methyltransferase	CG site maintains methylation
			osa-miR398a^*^↑	Os02g0554000^***^↑	SDG725	Histone H3K36 methyltransferase	RdDM

Fold change: DEmiRs-DEMRs identified based on expression levels; SSN, DImiRs-DIMRs identified based on single-sample network analysis. "↑ " indicates upregulation of differentially expressed genes; “↓” indicates downregulation of differentially expressed genes. “*” represents P-value< 0.05, “**” represents P-value< 0.01 and “***” represents P-value< 0.001. The single sample T test.

A comprehensive study on gene expression and interaction levels at different growth stages of rice reveals multiple miRNAs predicted to target DNA methylation regulatory factors. During the tillering stage, osa-miR397b, osa-miR398a, osa-miR5083, and osa-miR5079a are predicted to target histone methyltransferases and demethylases involved in the RdDM pathway in rice. Additionally, osa-miR169a is predicted to target *RDR6*. osa-miR2863c and osa-miR5501 are predicted to target DNA maintenance methyltransferases and demethylases. During the heading stage, osa-miR398a, osa-miR160f-5p, osa-miR11339-5p, and osa-miR1320-5p are predicted to target histone methyltransferases. Meanwhile, osa-miR5159 and osa-miR1874-5p are predicted to target *AGO4a*, while osa-miR1850.2 is predicted to targets *RDR2*. Additionally, osa-miR166j-5p is predicted to target DNA methyltransferases involved in maintenance methylation.

## 4 Discussion

Due to the absence of Earth’s magnetic field protection, organisms participating in deep space exploration missions are subjected to elevated radiation levels of GCR and SPE. Rice seeds that had been exposed to lunar orbit stressors for 23 days with a cumulative radiation dose of 59.85 mGy, were sown and subjected to phenotypic assessment. We noted a survival rate of merely 43% among the returned samples, indicating significant deleterious effects on the plant seeds under lunar exploration conditions. To deepen our understanding of the intrinsic molecular alterations prompted by lunar orbit flight in rice plants, we randomly selected three plants each at the tillering and heading stages, and collected leaf samples for transcriptome and DNA methylation sequencing. Here, we performed an integrated analysis of DNA methylation and transcriptome data, revealing the methylation and transcriptomic differences in rice under lunar orbital stressors. Additionally, due to the unique space environment, we conducted methylation and transcriptome analyses on each individual rice sample. We found that while the specific stress-responsive genes varied among individual rice plants, their biological functions were largely consistent. We also identified a group of differential miRNAs that may target MRs.

### 4.1 Effects of lunar orbit stressors on DNA methylation and transcriptome of rice (inter-group analysis)

DNA methylation is a crucial epigenetic modification that plays a significant role in rice growth and development. Through whole-genome bisulfite sequencing of rice leaves at the tillering and heading stages, we found that lunar orbital flight induced a reduction in the average methylation levels of CG, CHG, and CHH, but it was not significant (**Figure 1A**). Xu et al. found that the methylation levels in the CG, CHG, and CHH contexts of the Arabidopsis genome were lower under spaceflight conditions (Xu et al., 2018). Studies have also shown that plants exhibit non-significant changes in genome-wide methylation levels in response to stress (Zhou et al., 2019; Sun et al., 2021). It has been demonstrated by Xu et al. that spaceflight-induced differential DNA methylation exhibits transgenerational inheritance effects (Xu et al., 2021).

The GO enrichment results (**Figures 2D**, **3B, E**) indicate that DMGs and DEGs are primarily involved in metabolic processes, response to stimulus, and transport processes. The metabolic processes include organic substance metabolic processes, lipid metabolic processes, and primary metabolic processes, among others. Multiple studies have demonstrated that plants grown in spaceflight undergo common processes of cell wall remodeling, oxidative stress, defense responses, and photosynthesis alterations (Manzano et al., 2022). Olanrewaju et al. have demonstrated an increase in metabolic energy demand in plants within space environments, leading to the diversion of various metabolic pathways, including carbon metabolism, glycolysis, gluconeogenesis, and amino acid biosynthesis (Manzano et al., 2022; Olanrewaju et al., 2023). Overall, the findings of this study align with previous research on near-Earth space flights (such as SJ-10, ISS), indicating disruptions in plant oxidative stress, metabolic, and related pathways in spaceflights (Sugimoto et al., 2014; Choi et al., 2019; Chandler et al., 2020; Kruse et al., 2020; Zeng et al., 2020). DNA methylation has been demonstrated to play a crucial role in gene regulation (Zilberman et al., 2006; Stroud et al., 2013; Zhou et al., 2019; Pan et al., 2022). Zhou et al. have demonstrated that the changes in expression of stress genes, such as those involved in oxidative stress and defense signaling pathways in Arabidopsis induced by spaceflight, are due to alterations in DNA methylation (Zhou et al., 2019).

We also observed that the lunar orbital stressors induced changes in DNA methylation levels in the promoter regions of certain stress-responsive genes, potentially leading to alterations in their expression levels. These stress-related genes are largely involved in plant stress responses, including transcription factors *OsWRKY76* and *OsDREB1B*. For example, *OsWRKY63* acts as a transcriptional repressor, downregulating the expression of many genes associated with cold stress and reactive oxygen species scavenging (Zhang et al., 2022). The overexpression of *OsWRKY76* has been shown to suppress the induction of pathogenesis-related genes and phytoalexin biosynthesis genes, while enhancing the expression of genes related to abiotic stress, such as peroxidase and lipid metabolism genes. As a result, *OsWRKY76* plays a dual and opposing role in resistance to rice blast and cold tolerance (Yokotani et al., 2013). *DREBs/CBFs* transcription factors are capable of specifically binding to the DRE/CRT cis-elements, regulating the expression of numerous stress-induced genes (Dubouzet et al., 2003; Gutha and Reddy, 2008). Additionally, several transporter genes, such as *OsPT2*, *OsMTP8.1*, and *OsZIP1*, were identified. Our data indicate that the methylation level of the *OsPT2* promoter decreased, while the promoter methylation levels of the metal transporter genes *OsMTP8.1* and *OsZIP1* increased. The overexpression of *OsMTP8.1* and *OsZIP1* was found to prevent the excessive accumulation of zinc, copper, and cadmium in rice plants, thereby promoting healthy growth and development (Chen et al., 2013; Liu et al., 2019). Differential genes were also identified in the plant hormone-mediated signaling pathways. For example, the expression levels of the *OsGA2ox10* and *OsABA8ox2* genes were found to be affected. The increased expression level of the *OsGA2ox10* gene, induced by Lunar Orbit Stressors, is likely due to a decrease in promoter methylation. Gibberellin 2-oxidases (*GA2oxs*) regulate plant growth by deactivating biologically active endogenous gibberellins (GAs) (Lo et al., 2008). Abscisic acid (ABA) plays a crucial role in the regulation of seed germination and post-germination development (Zhu et al., 2009). The *OsABA8ox2* gene is involved in the catabolism of ABA, influencing the material and energy metabolism in rice seeds, which subsequently leads to changes in dormancy (Zhang et al., 2020; Fu et al., 2022).

Additionally, it was found that these genes are involved in biological pathways such as lipid metabolism and sugar metabolism. For example, the demethylation of the CG region in the promoter of *OsFAH1* may lead to an upregulation of its expression. *OsFAH1* is capable of catalyzing the 2-hydroxylation of sphingolipid fatty acids, thereby participating in the metabolism of reactive oxygen species induced by spaceflight (Nagano et al., 2016). The differentially expressed genes *OsUXS5* and *OsSWEET2a* encode UDP-xylosyltransferase and hexose transporter, respectively, and they regulate the response of rice to the stressor of lunar orbital pressure by participating in sugar metabolism (Suzuki et al., 2004; Yang et al., 2023). In summary, it has been found that most of the stress-related genes, whose expression levels may be altered due to changes in DNA methylation levels, are involved in the plant stress response. However, no specific class of genes has yet been identified that can clearly represent the stress response to spaceflight, which may be attributed to the complexity of the space environment.

Furthermore, the functional genes and MRs exhibited dynamic changes from the tillering stage to the heading stage. Studies have reported that patterns of transcription and DNA methylation vary across different developmental stages of rice. For instance, Basu et al. identified differential expression patterns of genes involved in regulating ion homeostasis across various developmental stages of rice (Basu et al., 2020). And DNA methylation profiles of SPLs and MIR156s exhibit distinct methylation patterns across different growth stages of rice (Yue et al., 2021). Additionally, expression of starch biosynthetic proteins in rice seeds varies across different developmental stages (Tappiban et al., 2021). Zeng et al.’s study also indicated that spaceflight-induced proteins in rice exhibit specificity at different growth stages, with signaling processes predominantly concentrated during the three-leaf stage and metabolic processes predominantly concentrated during the tillering stage (Zeng et al., 2020). Therefore, we believe that genes exhibiting different expression patterns across various developmental stages are a normal requirement for plant growth, development, and stress resistance.

### 4.2 The necessity of single sample analysis

As is well known, within the scope of the same space mission, plants will also be exposed to the influence of different types and doses of radiation particles. For example, in experiments conducted aboard the SJ-10 returning satellite orbiting at an altitude of 252 kilometers, research revealed that non-hit seeds comprised approximately 13% of the total, while the remaining seeds were subjected to impacts ranging from 1 to 8 times (Zhou et al., 2018). Therefore, during the same flight, plants are exposed to varying types and doses of radiation particles, resulting in inconsistent levels of stress. Previous studies have shown that compared to the control group, Arabidopsis exposed to different radiation treatments (30, 60, 110, or 430 mGy/h) for 14 days exhibited different methylation levels (Laanen et al., 2021). Gombeau et al. found that exposure to environmental dose rates ranging from 0.4 to 2.8 μGy/h resulted in tree frogs absorbing dose rates of 0.3 to 7.7 μGy/h, exhibiting a dose-dependent increase in global DNA methylation levels (Gombeau et al., 2020). The type of radiation ions also influences the DNA methylation patterns in plants (Yu et al., 2011). Furthermore, DNA methylation plays a critical role in influencing both individual radiation sensitivity and adaptive responses. The radiation sensitivity of seedling individuals is linked to specific patterns of DNA methylation that are regulated and reorganized in response to radiation exposure (Kravets and Sokolova, 2020). Consequently, we utilized single sample analysis to further elucidate the transcriptomic and methylation alterations in individual rice plants following lunar orbital flight.

Space radiation can induce changes at multiple molecular levels within cells, including regulation of gene expression, protein modifications, activation of cellular signaling pathways, and adjustments in cellular metabolism (Kuijjer et al., 2019). Therefore, the biological effects induced by space radiation constitute a complex process, and relying solely on the expression levels of individual genes cannot comprehensively explain the response to space radiation (Zhang et al., 2024a). To gain a more precise understanding of the biological response to space radiation, we utilized the SSN method to analyze the transcriptome profiles and construct SSNs for each sample. Our previous research validated the robust performance of the SSN model in exploring gene interactions. By employing this method, we revealed the impact of spaceflight on mouse gene interaction patterns, demonstrating a dose-dependent effect of radiation exposure (Zhang et al., 2024b). Furthermore, we found that this model accurately predicts the individual absorbed radiation dose equivalents in space environments (Zhang et al., 2024a). In this study, in deep space, the oxidative stress response primarily driven by DEGs occurs mainly during the heading stage, whereas the time points at which DIGs drive this process include both the tillering and heading stages (**Figures 5E, F**). This indicates that analyzing both gene expression and gene interaction levels simultaneously provides a more comprehensive understanding of the biological changes induced by lunar orbit stressors.

### 4.3 Effects of lunar orbit stressors on DNA methylation and transcriptome in different rice individuals

To further understand impact of lunar orbit stressors on genes and DNA methylation of different rice individuals, we conducted GO analysis on DEGs, DIGs, and DMGs (**Figures 4C, D**, **5E, F**). The results revealed that while the DEGs, DIGs, and DMGs generated by different rice individuals exhibited specificity, they were largely involved in consistent biological processes at the transcriptome and DNA methylation levels. Moreover, these genes primarily participate in metabolic regulation.

In both inter-group and single-sample analysis, we observed changes in the methylation patterns of rice after lunar orbit flights. There is partial overlap between differential genes in transcription level and methylation level, primarily involved in biological functions such as metabolism, response to stimuli, DNA damage response, and the cell cycle (**Figure 3E**; **Supplementary Figure 3C**). However, through single-sample analysis, we also identified certain DEmiRs (DImiRs) and DEMRs (DIMRs) with targeting effects, which are crucial for understanding the impact of miRNA on methylation under spaceflight conditions. Therefore, through inter-group analysis and individual plant analysis, we can comprehensively observe the lunar orbital stressors’ impact on rice and ensure the reproducibility of the results.

MRs are potential drivers of DNA methylation alterations (Ou et al., 2009). Therefore, we further investigated MRs in different rice individuals exposed to lunar orbit flight. The results revealed significant changes in the expression and interaction patterns of MRs during DNA methylation maintenance, demethylation, and RdDM. We utilized the psRNATarget online tool to predict differential miRNAs targeting MRs. The results indicate that predicted miRNA-MR target pairs exhibit differences among different rice individuals. However, these differential miRNA predictions predominantly target DNA methyltransferases at CG sites and genes associated with the RdDM pathway in plants. Specifically, during the tillering and heading stages, miRNAs such as osa-miR2863c and osa-miR166j-5p may target the *MET1b* gene. *MET1b* is a CG site maintenance methyltransferase in plants (Yamauchi et al., 2008). Furthermore, miRNA predictions target genes involved in the RdDM pathway, such as *SDG* family members, *JMJ706*, as well as *AGO4*, and *RDR2/6* (Yamauchi et al., 2008; Qin et al., 2010; Chen and Zhou, 2013; Sun et al., 2021). Moreover, Homocysteine is converted back into methionine by *HMT*, thus completing the cycle and replenishing the reservoir of stable methyl donors essential for maintaining DNA methylation patterns (Bradbury et al., 2014; Huang et al., 2022; Lee et al., 2023). During the tillering stage, miRNA predictions target *HMT* and DNA demethylase (*ROS1c*) (Sun et al., 2021; Zhou et al., 2021). The targets identified in this study provide a basis for further experimental validation.

Moreover, changes in methylation levels are attributed to a combination of multiple mechanisms. Firstly, repair of broken chromosomes results in reduced DNA methylation in plants (Yu et al., 2011). Additionally, oxidative damage can also lead to changes in methylation patterns (Yang et al., 2008; Yu et al., 2011). Lastly, ionizing radiation may induce changes in DNA methylation through chemical modifications of cytosine, mutations at methyltransferase recognition sites, or mutations affecting the regulation of gene expression (Yu et al., 2011). Our findings merely suggest that miRNAs may influence changes in methylation regulatory factors. It is far from comprehensive to suggest that the global genomic methylation levels in rice are solely regulated by methylation regulatory factors.

## 5 Conclusion

**Figure 7:**
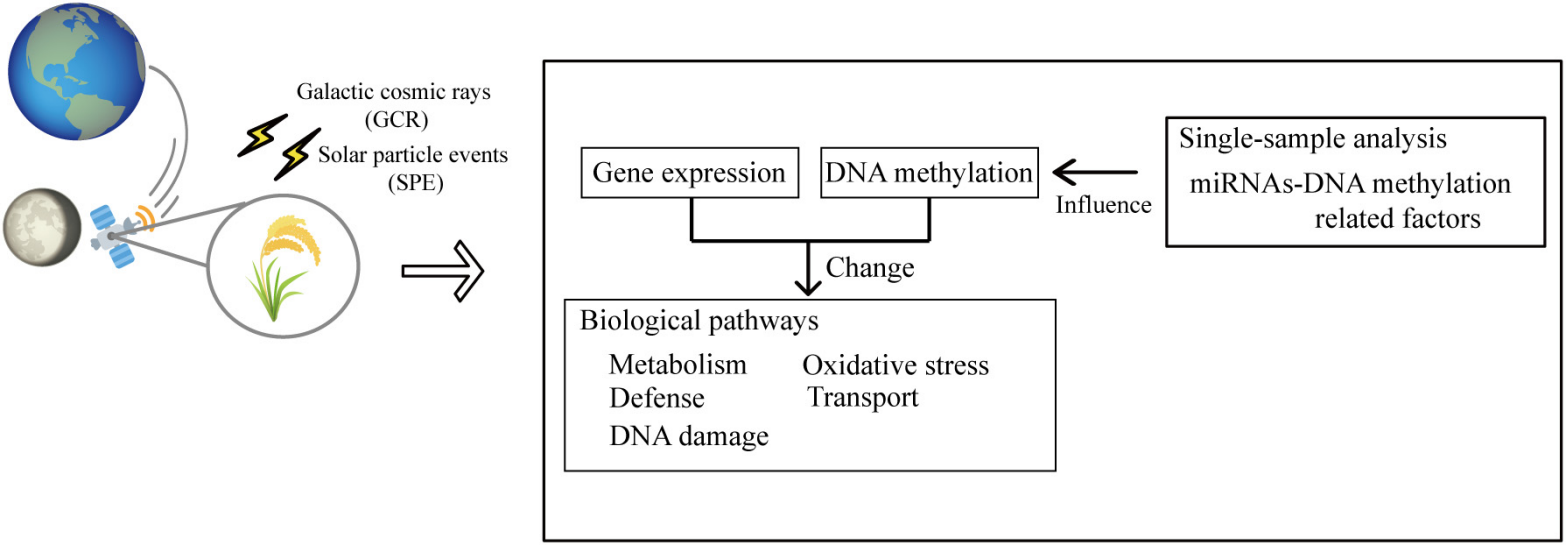
Changes in DNA methylation and the transcriptome of rice were induced by lunar orbital stressors.

The study indicates that the methylation patterns in the rice genome exhibit variability in response to lunar orbital stressors. DNA methylation alters the expression and interaction patterns of functional genes, involving biological processes such as metabolism and defense. Furthermore, through single-sample analysis, we identified a set of miRNAs induced by lunar orbital stressors. These miRNAs potentially target DNA methylation regulatory factors (**Figure 7**).

## Data Availability

The raw sequencing data generated in this study (WGBS and whole transcriptome sequencing) are available in the Genome Sequence Archive under the identifiers CRA013973 and CRA018677 (https://ngdc.cncb.ac.cn/gsa/). The expression levels of mRNA and miRNA can be found in the **Supplementary Information**.
