# Supplementary material for: Variations in DNA methylation and the role of regulatory factors in rice (Oryza sativa) response to lunar orbit stressors
Source: Front Plant Sci. 2024 Nov 14;15:1427578. doi: 10.3389/fpls.2024.1427578 (PMC11603183; doi:10.3389/fpls.2024.1427578)
Supplement: Supplementary file 1 [file DataSheet1.docx]

Supplementary Material

# Supplementary Figures and Tables

## Supplementary Figures

**Supplementary Figure 1.** Single-sample analysis of methylation changes in upstream, downstream, and functional regions of genes during the tillering and heading stages in rice. (A). DNA methylation profiles of mCG, mCHG and mCHH surrounding genes (within 2kb) in rice. (B). DNA methylation profiles of mCG, mCHG, and mCHH in gene functional regions of rice. *TC: Ground control group during the tillering stage, n=3; TS1/TS2/TS3: Spaceflight group during the tillering stage, n=1; HC: Ground control group during the heading stage, n=3; HS1/HS2/HS3: Spaceflight group during the heading stage, n = 1.*

**Supplementary Figure 2.** The tillering and heading stage DEGs intersect.

**Supplementary Figure 3.** Single-sample analysis of DMGs and DEGs. (A). Overlap of differentially methylated genes (DMGs) among different rice samples. (B). The Venn figure illustrates the association between three contexts of DMGs and spaceflight induced DEGs during the tillering and heading stages of different rice individuals. (C). GO analysis of overlapping genes between DMGs and DEGs (P-value < 0.05). The left figure displays GO enrichment analysis of overlapping genes between DMGs and DEGs (P-value < 0.05), while the right figure illustrates GO enrichment analysis of metabolic processes among overlapping genes between DMGs and DEGs (P-value < 0.05).

**Supplementary Figure 4.** DEmiR-DEMR and DImiR-DIMR network figure. *Triangles represent differentially expressed miRNAs (DEmiRs), with red indicating miRNAs overlapping between DEmiRs and DImiRs, orange indicating DImiRs, and yellow indicating DEmiRs; ellipses represent differentially expressed methylation regulators (MRs) targeted by these miRNAs.*

**Supplementary Figure 5.** Involvement of miRNAs in MRs of different rice individuals under lunar orbit stressors. *Purple font represents DEmiR-DEMR, and green font represents* *DImiR-DIMR.*

## Supplementary Tables

**Supplementary Table 1.** mRNA gene expression level.

**Supplementary Table 2.** miRNA expression level.

**Supplementary Table 3.** miRNA and Target Gene List.

**Supplementary Table 4.** List of primers used for qRT-PCR and stem-loop RT-PCR.

**Supplementary Table 5.** Gene list MRs in rice.

**Supplementary Table 6.** DEmiRNA and Target Gene List (n=3). *#N/A indicates non-DEmiRNA.*

**Supplementary Table 7.** Proportional classification of DMR locations. *TS1/TS2/TS3: Spaceflight group during the tillering stage, n=1; HS1/HS2/HS3: Spaceflight group during the heading stage, n = 1.*

**Supplementary Table 8.** List of DEMRs in Single Sample Analysis.

**Supplementary Table 9.** List of DIMRs in Single Sample Analysis.

**Supplementary Table 10.** List of DEmiRs in Single Sample Analysis.

**Supplementary Table 11.** List of DImiRs in Single Sample Analysis.

**Supplementary Table 12.** List of differential miRNAs and their target differential MRs identified using single-sample analysis method. *Fold change: DEmiRs-DEMRs identified based on expression levels; SSN: DImiRs-DIMRs identified based on single-sample network analysis; Fold change and SSN: Differences identified by both methods.*
