# Supplementary figures and images for: Variations in DNA methylation and the role of regulatory factors in rice (Oryza sativa) response to lunar orbit stressors
Source: Front Plant Sci. 2024 Nov 14;15:1427578. doi: 10.3389/fpls.2024.1427578 (PMC11603183; doi:10.3389/fpls.2024.1427578)

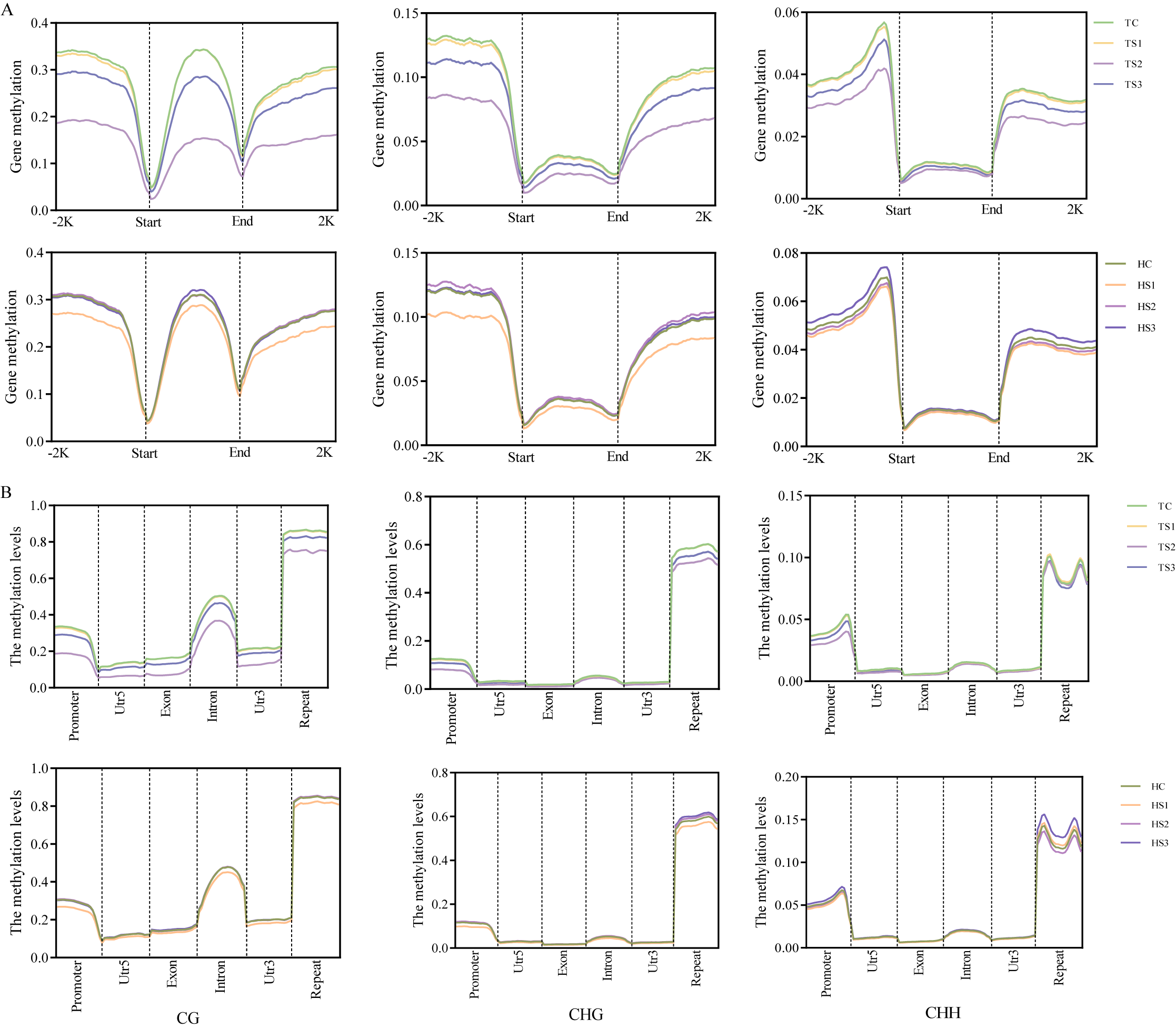

Supplement: Supplementary file 2 [file Image1.tif]

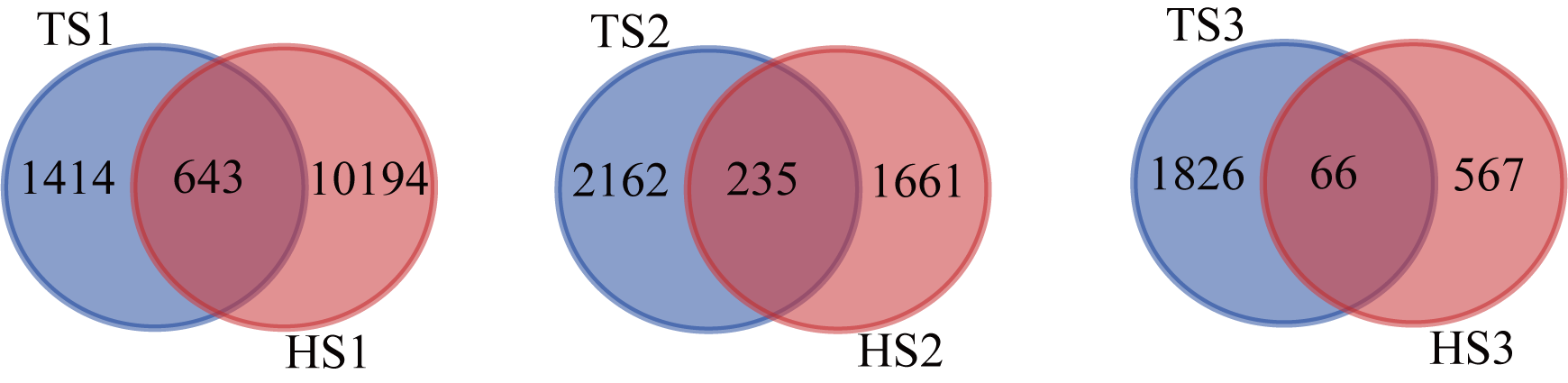

Supplement: Supplementary file 3 [file Image2.tif]

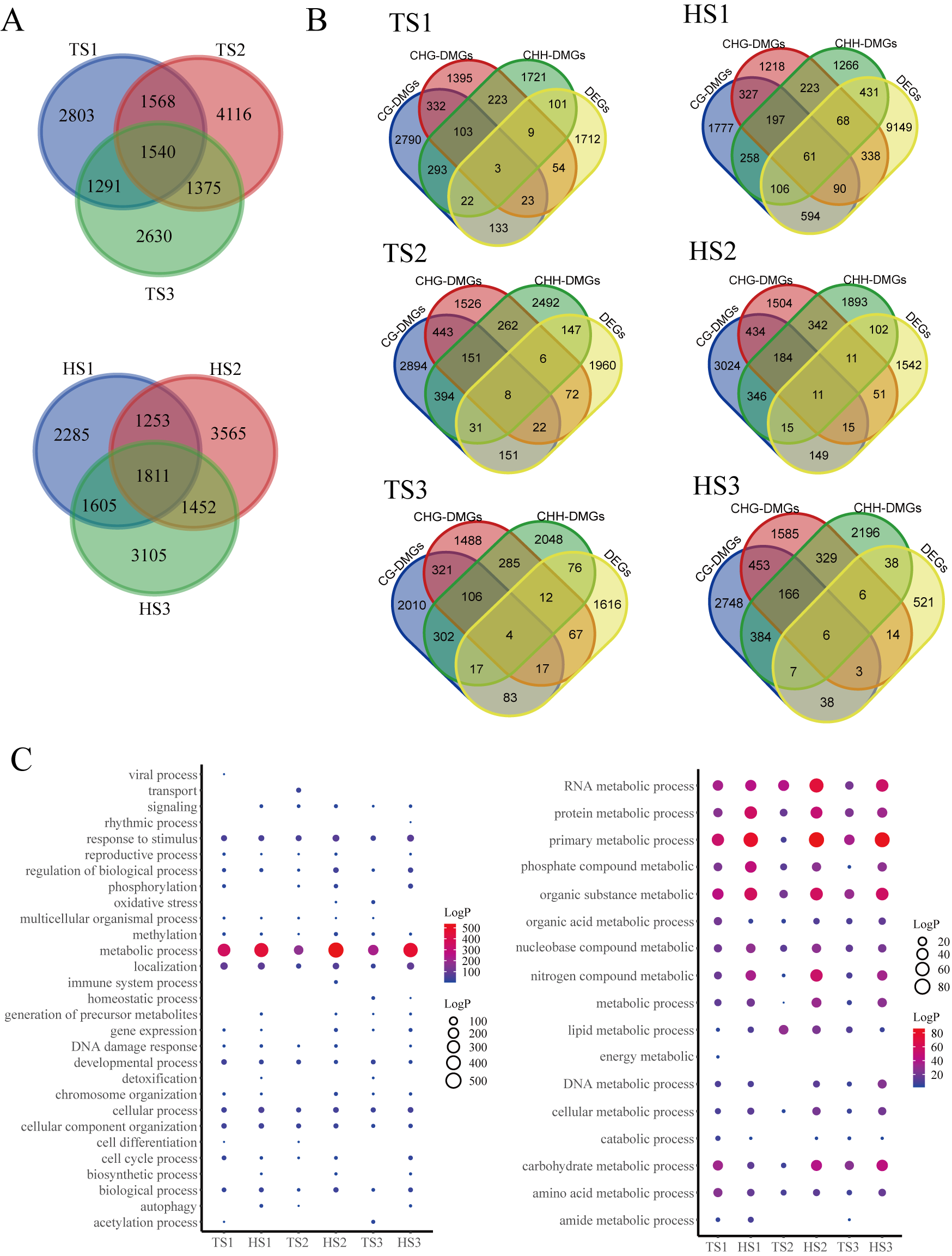

Supplement: Supplementary file 4 [file Image3.tif]

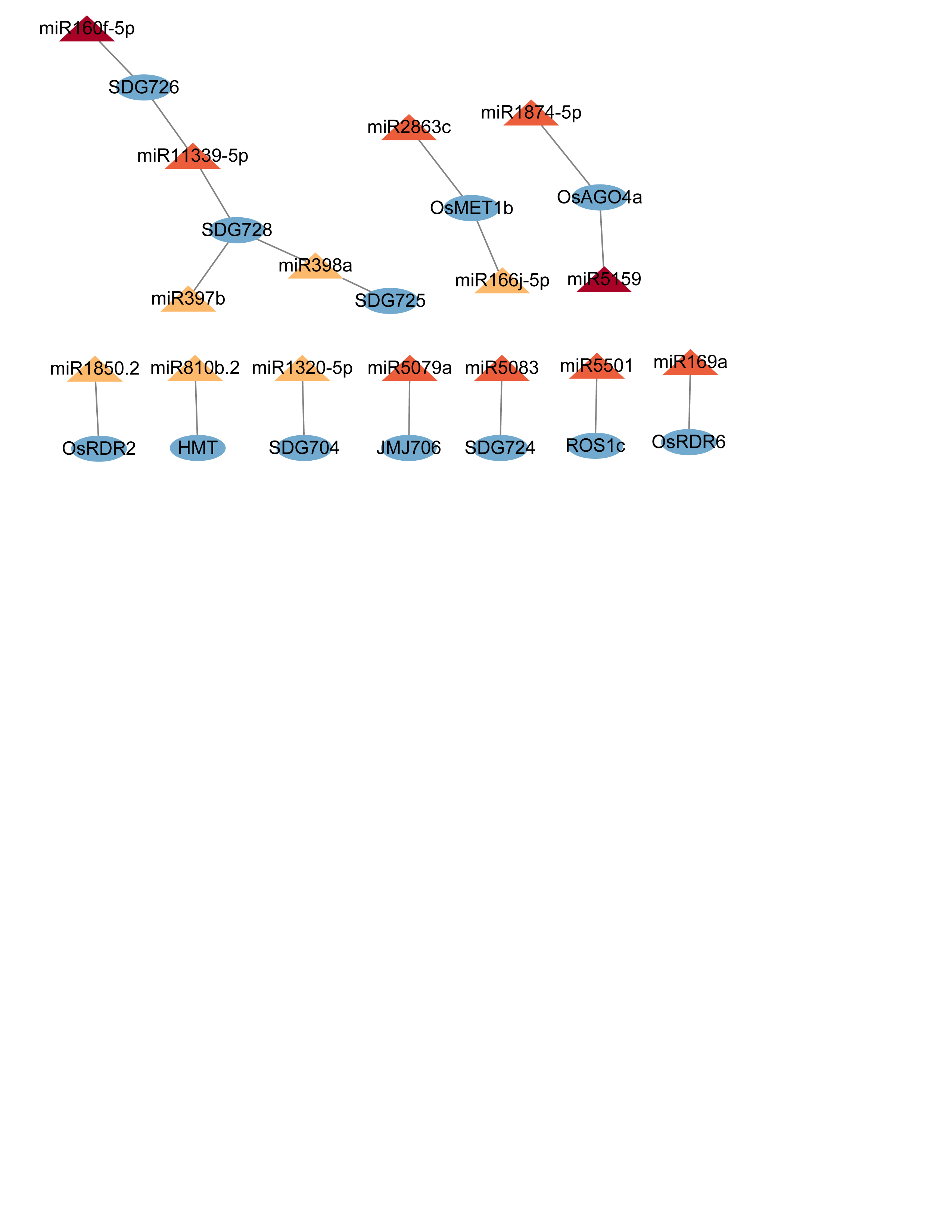

Supplement: Supplementary file 5 [file Image4.tif]

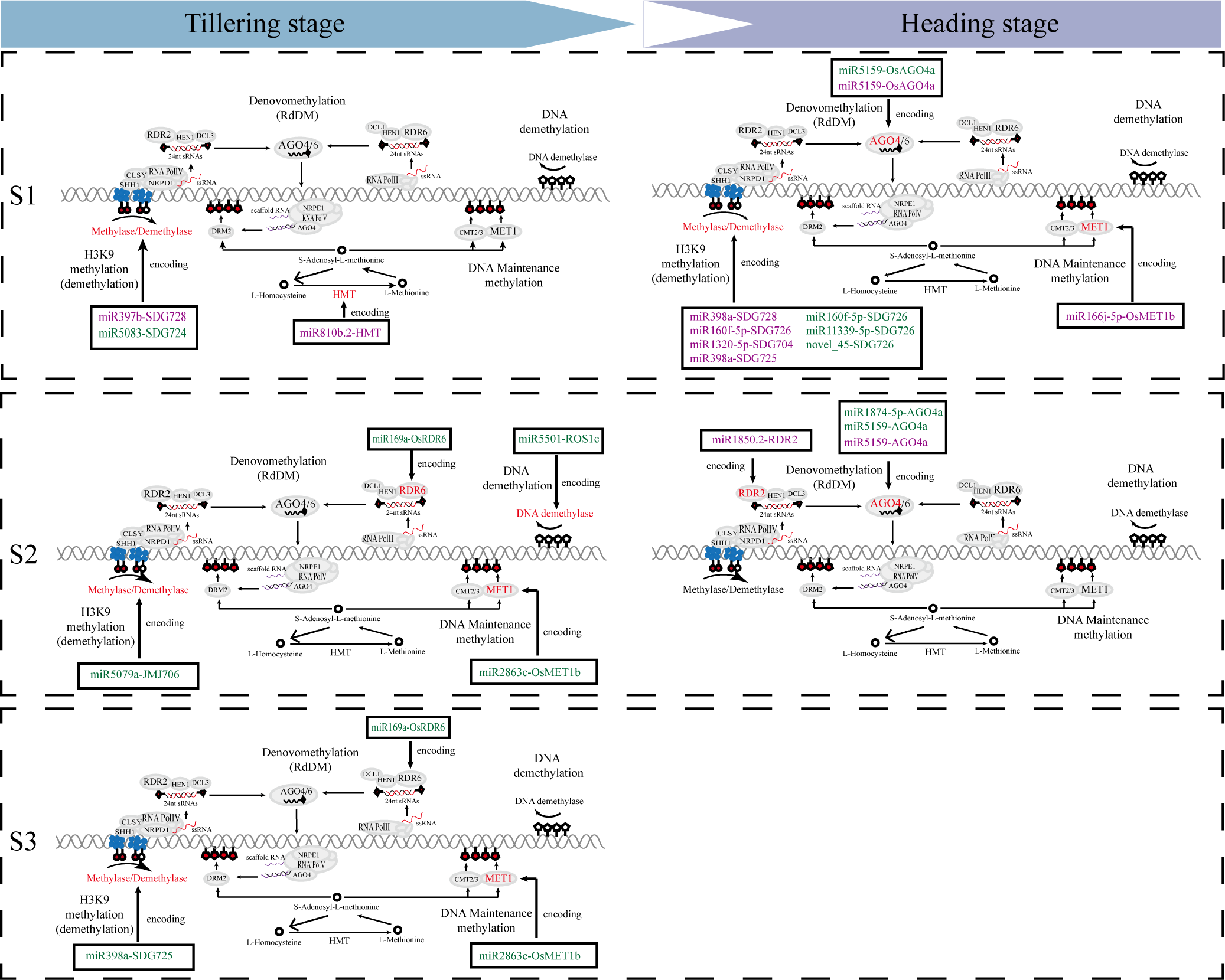

Supplement: Supplementary file 6 [file Image5.tif]
